# Draft Genome Sequence of Listeria innocua Strain MEZLIS29, Isolated from a Cow in South Africa

**DOI:** 10.1128/mra.01122-21

**Published:** 2022-02-28

**Authors:** Mohamed E. El Zowalaty, Alexandra Moura, Marc Lecuit, Oliver T. Zishiri

**Affiliations:** a Zoonosis Science Center, Department of Medical Biochemistry and Microbiology, Uppsala University, Uppsala, Sweden; b Institut Pasteur, National Reference Center and World Health Organization Collaborating Center for Listeria, Paris, France; c Institut Pasteur, Université de Paris, INSERM U1117, Biology of Infection Unit, Paris, France; d Necker-Enfants Malades University Hospital, Division of Infectious Diseases and Tropical Medicine, APHP, Institut Imagine, Paris, France; e Discipline of Genetics, School of Life Sciences, College of Agriculture, Engineering, and Science, University of KwaZulu-Natal, Durban, South Africa; University of Rochester School of Medicine and Dentistry

## Abstract

Here, we report the draft genome sequence of Listeria innocua strain MEZLIS29, which was isolated from a healthy cow in Flagstaff, Eastern Cape Province, South Africa. The genome was sequenced using the Illumina MiSeq platform and had a length of 2,805,865 bp, with a G+C content of 37.5% and 2,783 coding DNA sequences, 58 tRNAs, 4 noncoding RNAs, and 8 rRNA genes.

## ANNOUNCEMENT

*Listeria* species are small, motile, catalase-positive, non-spore-forming, rod-shaped, Gram-positive bacteria. The genus *Listeria* currently consists of 26 species (1), of which Listeria monocytogenes is a significant foodborne and invasive opportunistic pathogen causing serious epidemics and sporadic listeriosis in humans (2, 3). Listeria innocua is a nonpathogenic species that is closely related to L. monocytogenes (4). Although rare, atypical hemolytic L. innocua can actively cross the intestinal epithelium and spread systemically within the host (5). In addition to its ecological and clinical relevance (5–8), L. innocua genomes provide important insight into the evolution of pathogenicity of L. monocytogenes (4). Here, we report the draft genome sequence of L. innocua strain MEZLIS29, which was isolated from fecal material from a cow (Bos taurus) in Flagstaff, Eastern Cape, South Africa, in May 2018. The sample was analyzed as reported previously (9). The sample was collected in 10 mL of 0.1% buffered peptone water and incubated for 24 h. Following enrichment in *Listeria* broth (Oxoid, England), the sample was streaked onto *Listeria* selective agar (Oxoid) and incubated at 37°C for 18 h. A slant of the bacterial culture was shipped to the College of Veterinary Medicine, North Carolina State University, for further analysis. Colony PCR for the hemolysin (*hly*) gene was performed (10). An aliquot of overnight culture in brain heart infusion (BHI) broth was submitted to the Clinical Sciences Department at North Carolina State University for matrix-assisted laser desorption ionization–time of flight mass spectrometry (MALDI-TOF MS) using the VITEK MS identification system (bioMérieux, Marcy L’Etoile, France) and the associated software Knowledge Base v3.2 clinical US for further confirmation, as reported previously (9). The isolate was cultured on sheep blood agar for 18 to 24 h at 37°C in the presence of 5% CO_2_, and a single CFU was cultured in tryptic soy broth (TSB) to an inoculum density of a 0.5 McFarland turbidity standard. An aliquot of the culture in TSB was diluted with 0.85% sterile saline solution to the desired inoculum density of 1 × 10^6^ CFU/mL using a Thermo Fisher Scientific Sensititre nephelometer, and the chilled culture tube was submitted to the University of Minnesota Genomics Center for DNA extraction and whole-genome sequencing (WGS). DNA isolation was performed using a Qiagen DNeasy blood and tissue kit (Lucigen, WI, USA) according to the manufacturer’s protocol. The DNA sample was quantified using a fluorimetric PicoGreen assay using a Synergy 2 plate reader (BioTech). The purity of the DNA sample was also assessed using a Nanodrop 8000 instrument (Thermo Fisher).

**FIG 1:**
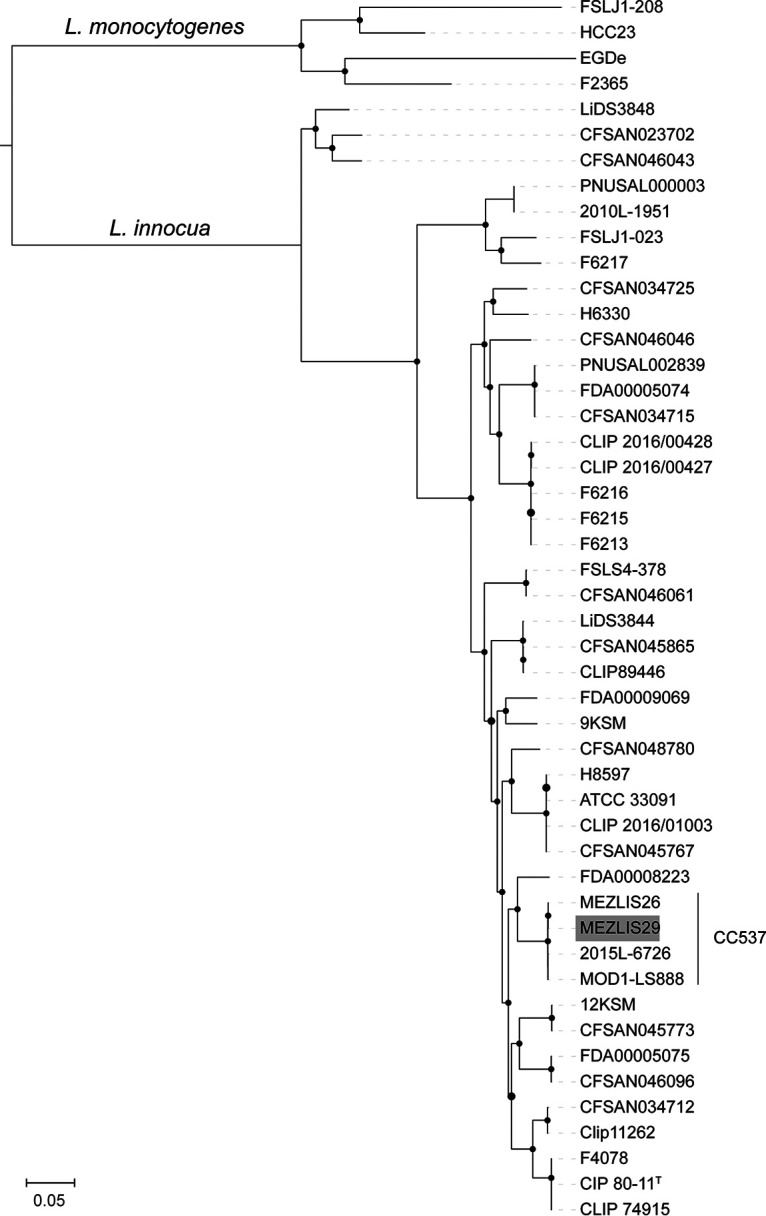
Positioning of isolate MEZLIS29 (highlighted in gray) within the L. innocua phylogeny. Representative genomes of L. monocytogenes were used as the outgroup. The maximum likelihood phylogeny was inferred from a core genome alignment of 632,995 nucleotide positions. Black circles represent bootstrap branch support values higher than 90%, based on 1,000 replicates.

Sequencing libraries were prepared using a Nextera XT library preparation kit (Illumina Inc., CA, USA). Sequencing was performed using the MiSeq platform (Illumina Inc., CA, USA) using the v2 reagent kit, which yielded 250-bp paired-end (PE) reads. Sequence analyses were performed at the National Reference Center and World Health Organization Collaborating Center for *Listeria* (Institut Pasteur, Paris, France). Raw PE reads (251,655 reads [54× coverage]) were trimmed for adaptors and low-quality bases with AlienTrimmer (11). Cleaned reads (247,781 reads) were assembled using SPAdes v.3.12.0 (12) using the automatic k-mer, --only-assembler, and --careful options. Contigs smaller than 300 nucleotides were discarded. Assembly quality was assessed using QUAST v.2.2 (13), yielding a total of 73 contigs and 2,805,865 bp, with a G+C content of 37.5% and an *N*_50_ value of 123,459 bp. The final sequence coverage of the assembled genome was 45×. The genome was annotated using the Prokaryotic Genome Annotation Pipeline (PGAP) v.5.1 (14) by the National Center for Biotechnology Information (NCBI), which identified 2,783 coding DNA sequences, 58 tRNAs, 4 noncoding RNAs, and 8 rRNA genes. A maximum likelihood phylogeny was inferred from the core genome alignment of 43 L. innocua and 4 L. monocytogenes public genomes (5) using Parsnp, implemented in the Harvest suite v.1.1.2 (15), and was visualized with iTOL v.4.2 (16). Isolate MEZLIS29 clustered within clonal complex 537 (CC537) (nonhemolytic L. innocua) (Fig. 1) together with isolate MEZLIS26 (GenBank accession number AADHQU000000000), which was previously isolated from a goat in the same region (9). The average nucleotide identity by BLAST of MEZLIS29 and MEZLIS26 was 100%, confirming the circulation of the same strain in different animal species.

### Data availability.

The Whole Genome Shotgun project has been deposited in DDBJ/ENA/GenBank under BioProject accession number PRJNA716986 (BioSample accession number SAMN18492169) and GenBank accession number JAGISK000000000. The version described in this paper is the first version, JAGISK000000000.1. The sequences have been submitted to the Sequence Read Archive (SRA) under the accession numbers SRX10515072 and SRR14145944. All isolates used in this study are also publicly available at https://bigsdb.pasteur.fr/listeria.
